# Dabrafenib- and trametinib-associated glomerular toxicity: A case report

**DOI:** 10.1097/MD.0000000000028485

**Published:** 2022-01-07

**Authors:** Eunmi Jo, Harin Rhee

**Affiliations:** a Department of Nephrology, Pusan National University School of Medicine, Busan, Republic of Korea; b Biomedical Research Institute, Pusan National University Hospital, Busan, Republic of Korea.

**Keywords:** BRAF inhibitor, dabrafenib, glomerular endothelial toxicity, mitogen-activated protein kinase inhibitor, melanoma, trametinib, vascular endothelial growth factor

## Abstract

**Rationale:**

Combined treatment with dabrafenib, a B-RAF inhibitor, and trametinib, a mitogen-activated protein kinase inhibitor, is an effective option for patients with metastatic melanoma. A few cases of acute kidney injury associated with tubulointerstitial nephritis and 1 case of nephrotic syndrome have been reported in patients on this drug combination; however, progressive renal injury has not been reported. In this case study, we report a patient with metastatic melanoma who developed glomerular capillary endothelial toxicity and progressive glomerular sclerosis during combination therapy.

**Patient concern:**

Our patient was an 80-year-old woman with a history of type 2 diabetes and chronic kidney disease.

**Diagnosis and intervention:**

She was diagnosed with metastatic melanoma and commenced combination therapy with dabrafenib and trametinib.

**Outcomes:**

Her renal function progressively deteriorated; by month 20 after treatment commencement, her serum creatinine level had increased from 1.59 to 3.74 mg/dL. The first kidney biopsy revealed marked glomerular and endothelial cell damage. Her medication was stopped, but no improvement was evident. At 5 months after the first biopsy, her serum creatinine level had increased to 5.46 mg/dL; a second kidney biopsy revealed focal segmental glomerular sclerosis and marked tubulointerstitial fibrosis. She was started on hemodialysis.

**Lessons:**

We describe a patient with a metastatic melanoma who developed progressive kidney failure during treatment with dabrafenib and trametinib. The most prominent microscopy findings were glomerular endothelial damage in the initial kidney biopsy and accelerated glomerular sclerosis and tubulointerstitial fibrosis in the follow-up biopsy. We hypothesize that a decreased renal reserve and impairment of kidney repair capacity caused by inhibition of B-RAF, a downstream mediator of vascular endothelial growth factor, may explain the progressive kidney injury.

## 1 Introduction

Metastatic melanoma is considered to have a poor prognosis, but patients with a BRAF V600E or V600K mutation enjoy prolonged progression-free and overall survival when treated with a combination of a B-RAF (BRAF) and a mitogen-activated protein kinase inhibitor.^[1]^ Combination treatment with dabrafenib and trametinib is an effective option not only for patients with metastatic melanoma but also for patients with nonsmall cell lung cancer or anaplastic thyroid cancer if the cancerous tissue harbors one of the mutations.^[2,3]^ A recent cohort study found that the incidence of acute kidney injury after combined dabrafenib and trametinib treatment was 21%^[4]^; however, the detailed phenotype of biopsy-proven nephrotoxicity remains poorly understood. One case of podocyte injury combined with clinically evident nephrotic syndrome has been reported,^[5]^ and a few case reports have suggested that the most common kidney injury is tubulointerstitial nephritis.^[5]^ In this case study, we report a patient with metastatic melanoma who developed glomerular capillary endothelial toxicity and progressive glomerular sclerosis during dabrafenib and trametinib combination therapy.

### 1.1 Case presentation

An 80-year-old woman visited our nephrology outpatient clinic for evaluation of gradual elevation of her serum creatinine (sCr) level. She had been diagnosed with malignant melanoma in 2010 and underwent amputation of her left third finger. In 2019, multiple subcutaneous nodules appeared on the left dorsal hand, accompanied by ipsilateral forearm and axillary lymph node enlargement. An excisional biopsy confirmed recurrent metastatic melanoma. As the cancer hosted the BRAF V600E mutation, she was prescribed combination therapy with dabrafenib (150 mg twice daily) and trametinib (2 mg once daily) She had a history of type 2 diabetes; her baseline sCr level was 1.59 mg/dL and her estimated glomerular filtration rate was 32 mL/min/1.73 m^2^ based on the Modification of Diet in Renal Disease equation.

**Figure 1:**
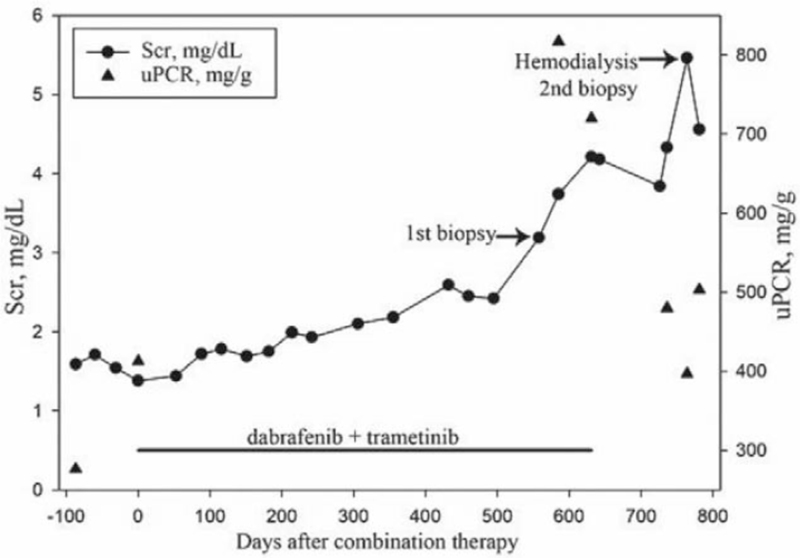
Changes in the serum creatinine level (mg/dL) and the urine protein/creatinine ratio during the course of disease. sCr = serum creatinine, uPCR = urine protein–creatinine ratio.

**Figure 2:**
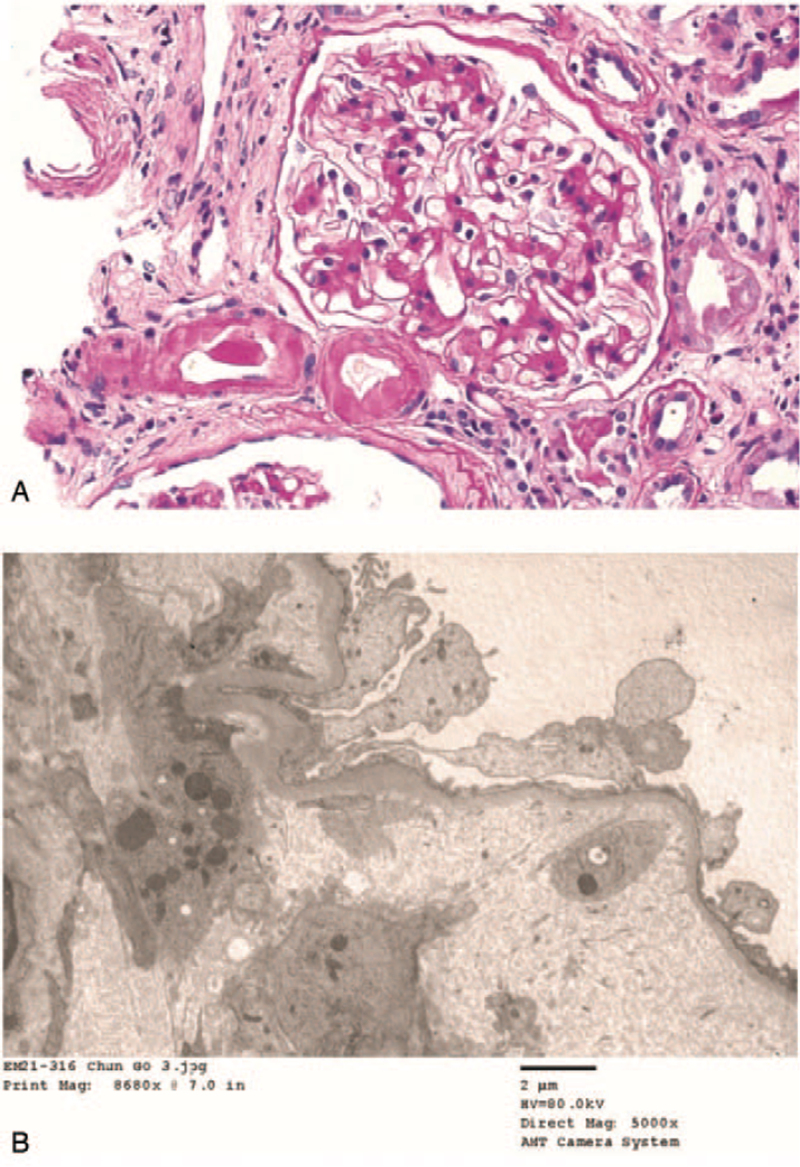
The first kidney biopsy findings. A. Light microscopy: the extent of the mesangial matrix is moderately increased. The glomerular capillary loops exhibit severe, subendothelial edematous widening; the lumina contain fluffy material. The capillary lumina are markedly reduced. The arterioles exhibit severe fibrinoid necrosis. Mesangiolysis is also evident. (H&E stain, ×400) B. Electron microscopy: the epithelial cell foot processes are widely effaced (×8680).

The combination therapy commenced in May 2019, and by 1 month later her renal function had begun to worsen (Fig. 1). By December 2020, her sCr level had gradually risen to 2.42 mg/dL. By March 2021, her sCr level had increased to 3.74 mg/dL and her urine protein/creatinine ratio had increased from 276 to 816 mg/g; she was therefore referred to our nephrology clinic. She denied any recent use of nonsteroidal anti-inflammatory drugs, proton pump inhibitors, or herbal medications. Given the temporal association between combination therapy and sCr elevation, we decided to perform a kidney biopsy. The serological parameters on the day of biopsy are summarized in Table 1. Light microscopy revealed 21 glomeruli, of which 4 (19%) exhibited global sclerosis. The remaining glomeruli were normocellular but slightly increased in size. The mesangial matrix was moderately increased. Ultrastructually, the glomerular capillary loops exhibited severe, subendothelial edematous widening, and the lumen contained fluffy material. The capillary lumina were markedly reduced and mesangiolysis was evident (Fig. 2A). No immunofluoresent staining was observed. Electron microscopy revealed widely effaced epithelial cell foot processes, and no electron-dense deposits (Fig. 2B). The final diagnosis was glomerular and arterial endothelial cell damage, possibly associated with anticancer therapy.

**Table 1:** Laboratory results at the time of kidney biopsy.

Parameter	Value	Reference range
Urine		
RBC, /HPF	0–2	0∼2
WBC, /HPF	16–20	0∼2
Protein (24 h), mg/d	297	40∼150
Protein (24 h), mg/dL	19.7	
Creatinine (24 h), mg/d	472	800–1800
Creatinine (24 h), mg/dL	31.3	
Protein/creatinine ratio, mg/g	816.87	150
Blood		
WBC, 10E3/μL	4.16	3.8∼11.0
Neutrophil, 10E3/μL	2.15	1.5∼7.0
Eosinophil, %	1	0∼6.0
Lymphocyte, %	38.3	20.0∼48.0
Hb, g/dL	10.6	11.2∼15.0
Platelet, 10E3/μL	266	140∼420
Albumin, g/dL	3.13	3.3∼5.2
BUN, mg/dL	40.5	6∼26
Creatinine, mg/dL	3.33	0.4∼1.2
Calcium, mg/dL	7.94	8.5∼10.3
Phosphorus, mg/dL	3.42	2.0∼4.6
Na, mmol/L	136.5	138∼148
K, mmol/L	4.65	3.5∼5.3
tCO_2_, mmol/L	19.6	20∼28
Uric acid, mg/dL	5.95	2.5∼8.0
CRP, mg/dL	0.62	0∼0.5
Anti-GBM Ab, U/mL	Negative: 2	Positve: >10
MPO Ab, U/mL	Negative (1.1)	Positive: ≥5.0
PR-3 Ab, U/mL	Negative (1.2)	Positive: ≥5.0
Anti-PLA2R IgG, RU/mL	Negative (<0.6)	Positive: ≥20
T. bilirubin, mg/dL	0.17	0.1∼1.2
Reumatoid factor, IU/mL	<7.0	0∼14
FANA	1:160	<=1:80
AST, U/L	20	0∼40
ALT, U/L	10	0∼40
ALP, U/L	120	35∼104
LDH, U/L	265	135∼214
IgG, mg/dL	1739	700∼1600
IgA, mg/dL	676	70∼400
IgM, mg/dL	233.6	40∼230
C3, mg/dL	106.2	90∼180
C4, mg/dL	24.1	10∼40

**Figure 3:**
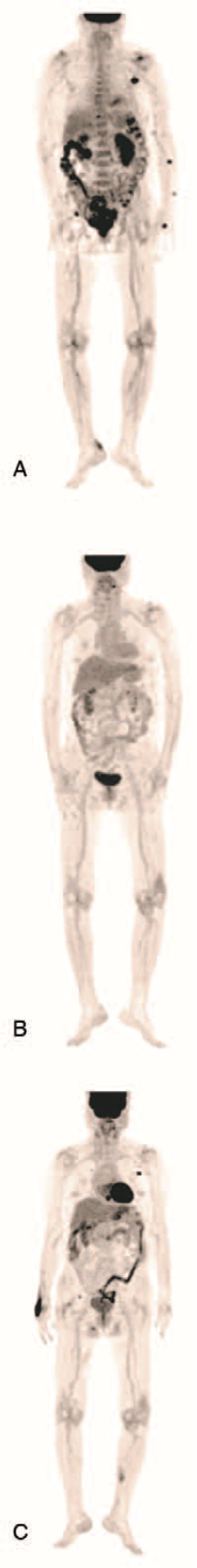
Positron emission tomography-computed tomography (PET-CT): A. at initial diagnosis of recurrent metastatic melanoma; B. 7 months after the commencement of combination therapy, a complete metabolic resolution is evident; and C. 5 months after stopping combination therapy, recurrence of melanoma is evident.

The metastatic melanoma responded well to combination therapy. By December 2019, she had achieved a complete metabolic response on positron emission tomography-computed tomography (Fig. 3). Given this encouraging disease status, both her oncologist and nephrologist agreed to stop the anticancer medications.

**Figure 4:**
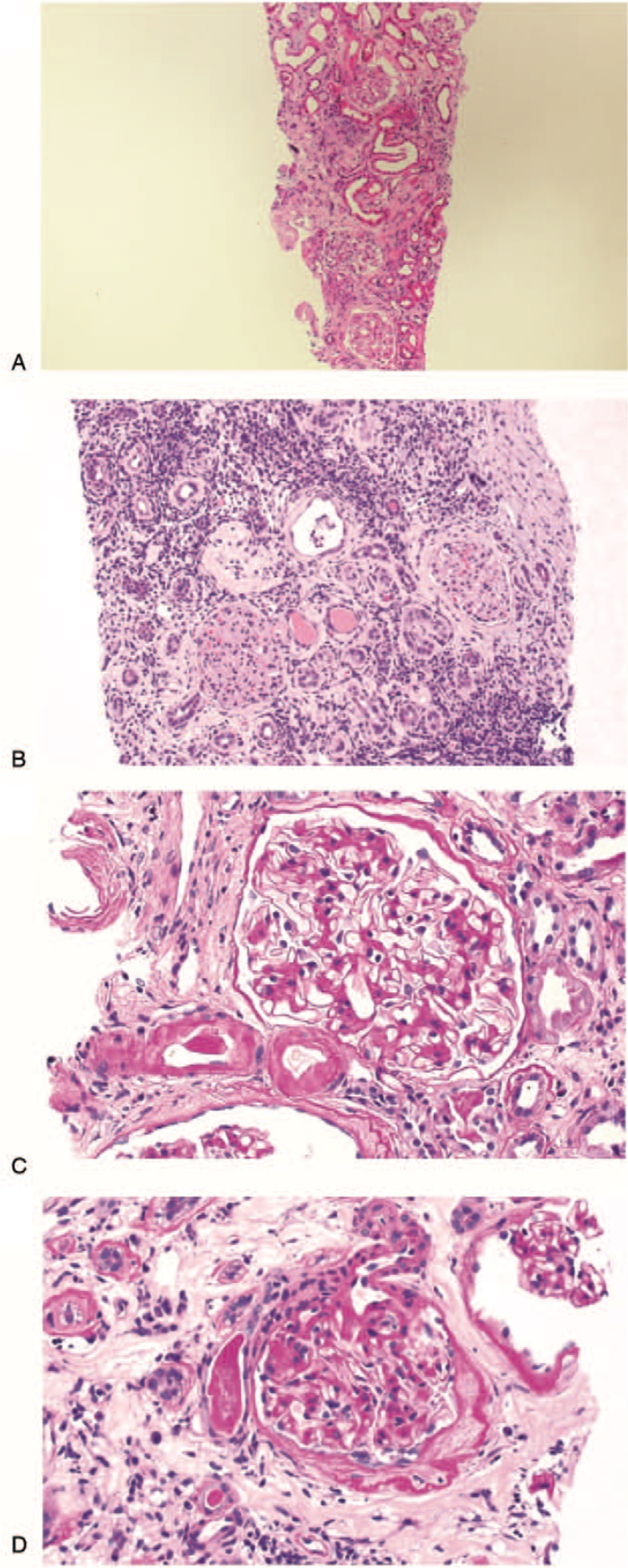
Comparisons between the initial and follow-up biopsy findings: A (initial) & B (follow-up) shows changes in tubules and interstitium. C (initial) & D (follow-up) shows changes in the glomerulus. The tubules exhibit severe focal atrophy with interstitial infiltration of mononuclear cells and fibrosis. Hyaline arteriolosclerosis is also apparent. Compared to the earlier biopsy specimen, the glomerular, vascular, and tubulointerstitial lesions are more advanced; they have become chronic.

At 2 months after cessation of combination therapy, sCr and proteinuria levels had decreased (Fig. 1). However, at 3 months later, a new melanoma metastasis was discovered on follow-up positron emission tomography-computed tomography, and second-line pembrolizumab was commenced. The patient immediately became uremic and her sCr level increased to 5.46 mg/dL. At 5 months after withdrawal of the initial drugs, thus 2 weeks after commencement of the new drug, a second kidney biopsy revealed marked increases in glomerulus, tubule, and interstitium disease chronicity (Fig. 4). The patient was thus started on hemodialysis.

## 2 Discussion and conclusions

We describe a patient with a metastatic melanoma who developed progressive kidney injury during treatment with dabrafenib and trametinib. The most prominent microscopy finding of the initial biopsy was glomerular capillary endothelial damage. On follow-up biopsy, the most obvious features were accelerated glomerular sclerosis and tubulointerstitial fibrosis. As endothelial cell injury is reported to play a role in progression of glomerular sclerosis, which is assumed to be the most important cause of progressive renal disease, we consider that the tissue findings were linked to the clinically observed progressive increase in her sCr levels. As suggested previously,^[6]^ suppression of angiogenesis after endothelial cell injury may inhibit regeneration of the glomerular architecture.

Diabetic nephropathy can also cause progressive kidney damage. Endothelial injury, mild mesangial expansion, and arteriolar hyalinosis are morphological hallmarks of diabetic nephropathy.^[7,8]^ However, the accelerated progression of glomerular sclerosis and tubulointerstitial fibrosis evident on examination of the second biopsy sample imply that the additional kidney damage was not attributable solely to diabetes. We hypothesize that the endothelial damage and accelerated tissue fibrosis were toxic effects of the anticancer drugs, superimposed on diabetic nephropathy. A decrease in the renal reserve caused by chronic diabetic nephropathy and impairment of kidney repair by a direct drug effect might have accelerated progression of kidney injury even after the drugs were stopped.

To date, 3 cases of tubulointerstitial nephritis^[4,9,10]^ and 1 case of nephrotic syndrome^[5]^ have been reported following combined treatment with dabrafenib and trametinib. An additional case of glomerulonephritis has been described, but this patient had been treated earlier with the immune checkpoint inhibitors ipilimumab and nivolumab.^[11]^ We found one similar case who received the immune checkpoint inhibitor pembrolizumab, but kidney biopsy data were lacking.^[12]^ Reports on BRAF–mitogen-activated protein kinase combination therapies using drugs other than dabrafenib and trametinib have indicated that acute tubular necrosis and tubulointerstitial nephritis are quite common,^[13–15]^ but only 1 case of glomerulonephritis combined with granulomatous vasculitis has been reported.^[16]^ To the best of our knowledge, this is the first reported case of direct glomerular toxicity causing progressive kidney injury associated with combination therapy (Table 2).

**Table 2:** Kidney biopsy findings d/t associated AKI.

Drug	Biopsy findings	Intervention	Kidney outcome	Ref
Dabrafenib + Trametinib				
Dabrafenib + Trametinib	Podocyte injury	Withholding the drugs	Complete recovery	^[4]^
Dabrafenib + Trametinib	Interstitial granulomatous nephritis	Withholding the drugs + corticosteroid	Complete recovery	^[6]^
Dabrafenib + Trametinib	Interstitial granulomatous nephritis	Withholding the drugs + corticosteroid	Improved but not quite to baseline	^[7]^
Dabrafenib + Trametinib	Acute tubular interstitial nephritis	Withholding the drugs + corticosteroid	Complete recovery	^[5]^
Dabrafenib + Trametinib^∗^	Glomerulonephritis + acute tubular nephritis	Withholding the drugs + corticosteroid	Improved but not quite to baseline	^[8]^
Other BRAF + MEK inhibitor				
Encorafenib + Binimetinib	Glomerulonephritis with granulomatous vasculitis	Withholding the drugs	Complete recovery	^[13]^
BRAF inhibitor monotherapy				
Vemurafenib	Acute tubular necrosis	withholding the drugs	Died 3 d after	^[14]^
Vemurafenib	Glomerulonephritis	Withholding the drugs + corticosteroid	Complete recovery	^[15]^

The glomerular toxicity of combination therapy may be more attributable to dabrafenib than to trametinib.^[17,18]^ Dabrafenib inhibits BRAF, a downstream signaling effector of vascular endothelial growth factor (VEGF). VEGF signals through the VEGF receptor, stimulating endothelial cell proliferation and migration via diverse intracellular signaling pathways: the mTOR, eNOS, and RAF/MAPK/ERK pathways. The RAF/MAPK/ERK pathway is affected by dabrafenib.^[19]^

In the glomerulus, VEGF is expressed and secreted by podocytes, and VEGF receptors are expressed on the surfaces of both endothelial cells and podocytes.^[20]^ Therefore, interruption of VEGF-mediated podocyte-endothelial crosstalk compromises the efficiencies of the glomerular filtration barrier and glomerular capillary endothelial cells.^[7,19]^

The pathological impacts of kidney VEGF inhibition can be seen in patients with pre-eclampsia.^[21,22]^ In pre-eclampsia, a soluble Fms-like tyrosine kinase-1 antagonizes VEGF and excess soluble Fms-like tyrosine kinase-1 triggers maternal endothelial dysfunction, hypertension, glomerular endotheliosis, and proteinuria; this combination of problems progresses to thrombotic microangiopathy.^[19,23,24]^ A similar spectrum of disorders has been noted after prescription of various VEGF inhibitors.^[24–28]^

The renal toxicities associated with BRAF inhibitor use include allergic interstitial disease, acute tubular necrosis, electrolyte imbalances, and proteinuria.^[29]^ In our case, the severe subendothelial edematous widening of the glomerular capillary loops and the foot process effacement were consistent with the previously reported glomerular toxicities developing after VEGF inhibition.^[24]^ The kidney injuries of previously reported acute interstitial nephritis cases seem to differ from those of our patient. The reported injuries appeared immediately (within 1–2 weeks) after drug initiation, and were probably an allergic reaction.^[29]^

In previous cases with interstitial nephritis or acute tubular necrosis, drug withholding induced complete or partial renal recovery. However, in our case, such withholding did not stop the kidney deterioration. A decreased kidney reserve, associated with pre-existing diabetic nephropathy and aging, may have played a role in the progression of kidney damage.

This case indicates that the risks and benefits of drugs must be balanced before they are withheld, especially when the drugs are associated with life-threatening cancers. In our case, melanoma recurred 5 months after cessation of the drugs.

Our case shows that long-term combined use of dabrafenib and trametinib can cause irreversible kidney injury through direct endothelial damage, especially in patients with pre-existing kidney disease. We suggest that when assessing the risks and benefits of combination therapy, oncologists should consider baseline kidney function before starting or stopping such medication.

## Author contributions

**Conceptualization:** Harin Rhee.

**Resources:** Harin Rhee.

**Supervision:** Harin Rhee.

**Validation:** Harin Rhee.

**Visualization:** Harin Rhee.

**Writing – original draft:** Eunmi Jo.

**Writing – review & editing:** Harin Rhee, Eunmi Jo.
